# Age-Period-Cohort analysis and 2036 projections of the burden of ischemic stroke in Finland, Korea, Singapore and China, 1990–2021

**DOI:** 10.3389/fneur.2025.1651799

**Published:** 2025-11-03

**Authors:** Jiayue Zhang, Bowei Zhao, Kai Wang, Lijing Zhao, Zhongxin Xu

**Affiliations:** ^1^Department of Neurology, China-Japan Union Hospital of Jilin University, Changchun, Jilin, China; ^2^Department of Rehabilitation Therapy, School of Nursing, Jilin University, Changchun, Jilin, China

**Keywords:** ischemic stroke, epidemiology, burden of disease, Bayesian Age-Period-Cohort model, disease prediction

## Abstract

**Objective:**

To analyze the temporal changes in ischemic stroke incidence and prevalence and their correlations with age, period, and birth cohort from 1990 to 2021 in Finland, Korea, Singapore and China, and to predict the trends of incidence and number of cases in 2036.

**Methods:**

The data from the Global Burden of Disease (GBD) 2021 database were used. The effects of age, period and birth cohort on the incidence of ischemic stroke were sorted out by age-period-cohort (APC) modeling. Bayesian Age-Period-Cohort (BAPC) modeling was used to predict the incidence rates of different sex and age groups in 2036.

**Results:**

The global all-age disability-adjusted life year (DALY) rate of ischemic stroke decreased by 34.90% between 1990 and 2021, with the highest disease burden in China, especially in males. Whereas, Singapore, South Korea, and Finland were lower than the global average. The APC model showed that the incidence increased significantly with the increase of age. Compared with China, the age of high incidence in Singapore, South Korea and Finland has moved forward. In terms of period effect, there were fluctuations across the countries. Although China is less affected by the cyclical effect, the overall burden is increased. In the cohort effect, the incidence of Singapore, Finland, and Korea showed a “U-shape,” while China's prevalence continued to decline. Projections for the next 15 years indicate that the incidence rates in Finland, Korea, and Singapore will remain low, whereas China may continue to increase. By 2036, it could reach nearly 200 per 100,000 people, and the uncertainty is greater, so it needs to focus on prevention and control.

**Conclusion:**

The burden of disease for ischemic stroke has been declining in all four countries from 1990 to 2021, but it has been rising in recent years and is expected to continue to rise over the next 15 years.

## Introduction

Ischemic stroke represents a major global public health challenge, characterized by high incidence, recurrence rates, disability, mortality and substantial economic costs (1). According to the Global Burden of Disease (GBD) 2019 study, ischemic stroke accounted for approximately 6.55 million deaths worldwide (2), establishing it as a leading cause of death and long-term disability, surpassed only by cardiovascular diseases and cancer (3). Although recent decades have seen advancements in prevention, diagnosis, and treatment, which led to declining mortality in some regions (4, 5), the overall burden of ischemic stroke continues to rise globally. This rise is largely driven by lifestyle changes and aging populations, particularly in developing countries (6, 7).

A thorough understanding of the evolving epidemiology of ischemic stroke is essential for facilitating early risk screening, acute treatment, sustained rehabilitation, and effective management of risk factors. It also supports policymakers in developing targeted strategies to alleviate pressure on healthcare systems.

Significant regional variations exist in the burden of ischemic stroke, reflecting differences in healthcare access, socioeconomic conditions, and geographic factors (2, 8, 9). For instance, countries such as Finland, South Korea, Singapore, and China differ considerably in economic development and health infrastructure, contributing to divergent trends in the prevalence and incidence of ischemic stroke (10–13). These differences underscore the need for country-specific analyses of disease burden.

Using data from GBD 2021, this study examines the temporal trends and geographical distribution of ischemic stroke in Finland, South Korea, Singapore, and China. In addition, we employ the Bayesian Age-Period-Cohort (BAPC) model to forecast incidence rates in these countries over the next 15 years. Our aim is to elucidate regional disparities in disease burden and contributing factors, thereby informing the development of tailored prevention and management strategies for ischemic stroke.

## Methods

### Data sources

This study is based on a secondary analysis of the GBD2021 dataset from the Global Health Data Exchange (GHDx) in Global Burden of Disease (GBD) covering 371 disease burdens in 204 countries and territories around the world from 1990 to 2021. The data was extracted on the fifteenth of November 2024. Over the subsequent month, the data underwent analysis and processing, culminating in the completion of the relevant charts.

The Global Burden of Disease (GBD) 2021 provides the distribution of diseases and injuries and their burdens over time, age, sex, and geography, while continuously updated databases allow for precise assessment of risk factor exposures, associations with health risks, and shares in the burden of disease (14). However, traditional epidemiological methods do not accurately analyze the correlates contributing to the disparities, whereas the BAPC model is based on an integrated nested Laplace approximation, which approximates the marginal posterior distributions and avoids the mixing and convergence problems introduced by the Markov chain Monte Carlo sampling technique traditionally used for Bayesian methods. The excellent predictive performance of the model has been verified. Therefore, the present study provides an in-depth analysis through the age-period-cohort (APC) model to clarify that these factors influence the epidemiology of ischemic stroke (15).

### Case definitions and data collection

In GBD 2021, ischemic stroke was defined using the International Classification of Diseases (ICD), including the 9th (ICD-9: 433-435.9, 437.0-437.2, 437.4-437.9) and 10th (ICD-10: G45-G46.8, I63-I63.9, I65-I66.9, I67.2- I67.848, I69.3-I69.4) (16). This study collected the data on the number of disability-adjusted life years (DALYs), all-age disability-adjusted life year (DALY) rates, and age-standardized DALY rates attributable to ischemic stroke globally and within the four countries listed above, covering age groups ranging from 0 to over 95 during the period 1990 to 2021. In this article, DALY is defined as the sum of the number of life years lost due to premature death from ischemic stroke and the number of life years lived in the disabled state, and is used to assess the burden of the disease on the overall population. The all-age DALY rate represents the total burden per year per population without age adjustment, calculated as the total number of DALYs in a given year divided by the total population during the same period. It reflects the crude disease burden affecting the entire population.

### Statistical methods and data analysis

All data were processed by R software (version 4.2.1). To estimate 95% uncertainty intervals (UI), the analysis was based on the intrinsic characteristics of the GBD database such as model selection, parameter estimation and data quality. These intervals were calculated by generating 1,000 samples after simulation, and the upper and lower limits correspond to the 2.5 and 97.5 percentiles of the results distribution, respectively. In addition, due to the data update of GBD 2021 version, the socio-demographic index (SDI) was introduced for these four countries as an important variable for the analysis in this study. The detailed methodology and modeling approach used in GBD 2021 were described in other publications (14, 17, 18). The data used in this study were anonymized and publicly accessible, and informed consent waiver approval was obtained from the University of Washington Institutional Review Board.

### SDI

The sociodemographic index (SDI) is a composite indicator developed by GBD researchers that is closely related to the burden of disease and health of a population. It calculates the geometric mean of three indices normalized between 0 and 1: the total fertility rate of the population under 25 years of age (TFU25), the average years of schooling of individuals aged 15 years and older (EDU15+), and the per capita lagged-distributed income (LDI) A value of 0 for the SDI represents the lowest level of development in terms of health-related aspects, while a value of 1 represents the most developed level. In the 2021 SDI, all countries were categorized into five categories based on SDI: low SDI: < 0.47; medium-low SDI: 0.47–0.62; medium SDI: 0.62–0.71; medium-high SDI: 0.71–0.81; and high SDI: > 0.81 (19). This study measures the level of development of these four countries through SDI.

### Age-period-cohort analysis

This study used age-period-cohort modeling analysis to assess the impact of age, period, and cohort effects on ischemic stroke incidence, prevalence, and DALY trends. The model independently estimates the coefficients of the effects of age, period, and cohort effects by using the Intrinsic Estimator (IE) subalgorithm. The IE method employs principal component regression techniques, and the robustness of its statistical properties has been validated in several modeling studies (20, 21). In this study, age was divided into one cohort for every 5 years of age, the period was divided into 6 cohorts (one cohort for every 5 years), and the data for the year in the middle of each cohort were used as the incidence of ischemic stroke during that period (22). A total of 25 birth cohorts were obtained (see Supplementary Table 1 for a detailed schematic). Incidence, prevalence and relative risk (RR) of DALY were obtained by transforming the natural logarithm of the effect coefficients. Finally, age-period-cohort analysis was performed using the Age Period Cohort Analysis Tool. Additionally, Bayesian Age-Period-Cohort (BAPC) (Version number: 0.0.36) analysis was performed in R language using BAPC and Integrated Nested Laplace Approximations (INLA) (Version number: 25.02.10) packages to predict the prevalence rates for different gender and age groups from 2022 to 2036.

## Results

### DALYs for ischemic stroke in 4 countries in 2021

**Figure 1:**
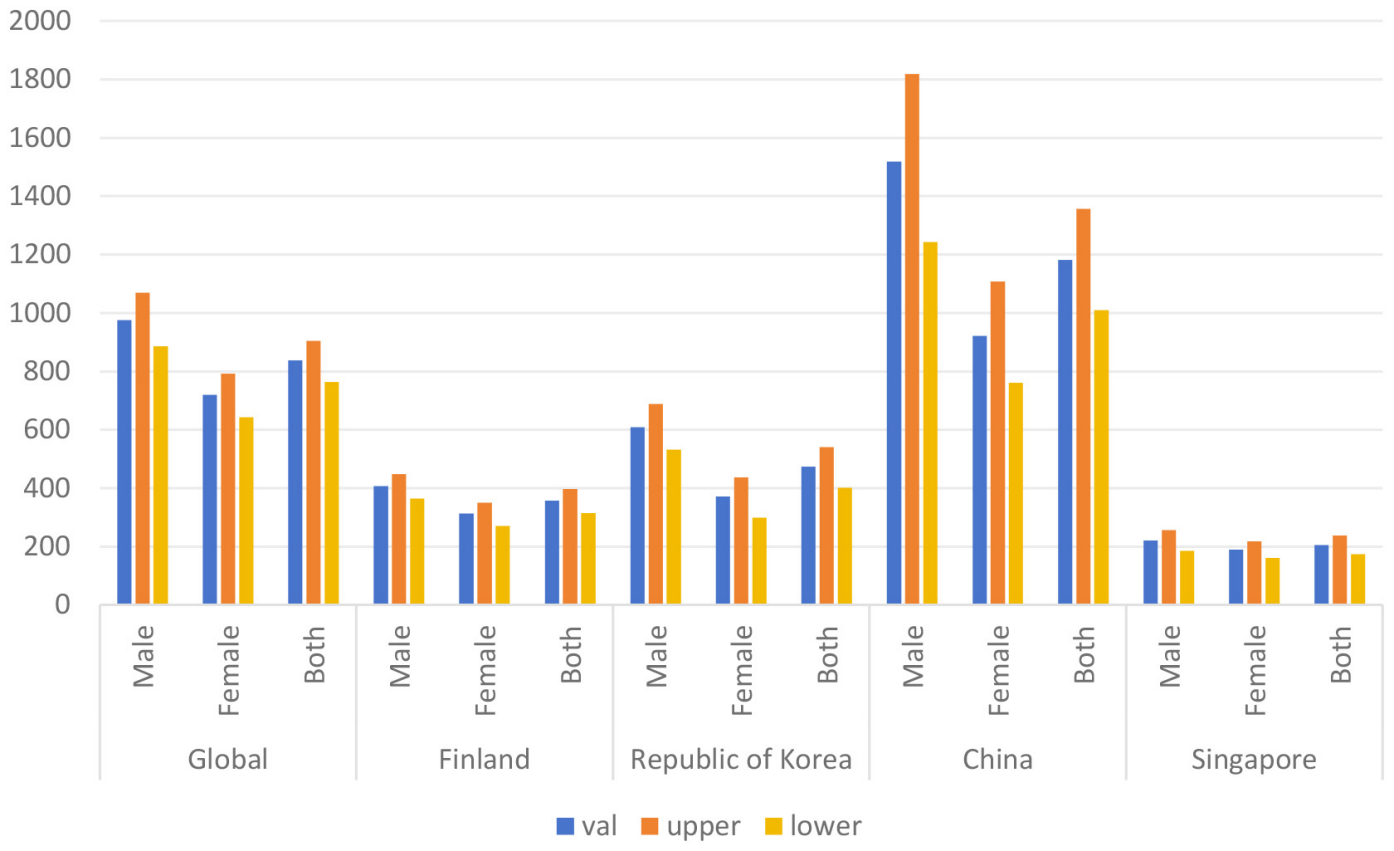
DALYs by all age groups globally and in China, South Korea, Finland, and Singapore.

In 2021, the global rate of all-age DALYs for ischemic stroke was 837.36 (95% UI 763.73–904.98) per 100,000 people, which was a reduction from 1,286.31 per 100,000 people (95% UI 1,195.19–1,376.06) in 1990. And of the 4 countries of interest, China had the highest ischemic stroke burden, with an overall level of 1180.98 (95% UI 1009.70–1356.67) per 100,000 people, especially for the male group of 1518.49 per 100,000 people. The DALY value for Singapore was relatively low, with an overall level of 205.84 (95% UI 174.50–237.16). And excluding China, the rates of DALYs in the remaining 3 countries were significantly lower than the global average (Table 1; Figure 1).

**Table 1:** DALYs for ischemic stroke in 4 countries in 2021.

**Location_name**	**Sex_name**	**Val**	**Upper**	**Lower**	**UI**
Global	Male	975.30	1069.81	885.61	975.30 (885.61, 1069.81)
	Female	719.52	791.64	642.82	719.52 (642.82, 791.64)
	Both	837.36	904.98	763.73	837.36 (763.73, 904.98)
Finland	Male	406.70	448.27	364.40	406.70 (364.40, 448.27)
	Female	312.94	349.48	269.98	312.94 (269.98, 349.48)
	Both	357.84	397.54	314.38	357.84 (314.38, 397.54)
Republic of Korea	Male	608.95	688.27	532.61	608.95 (532.61, 688.27)
	Female	371.47	436.46	298.91	371.47 (298.91, 436.46)
	Both	473.65	540.18	401.60	473.65 (401.60, 540.18)
China	Male	1518.49	1819.06	1243.01	1518.49 (1243.01, 1819.06)
	Female	921.94	1107.52	760.75	921.94 (760.75, 1107.52)
	Both	1180.98	1356.67	1009.70	1180.98 (1009.70, 1356.67)
Singapore	Male	220.69	256.34	185.50	220.69 (185.50, 256.34)
	Female	189.60	217.58	160.58	189.60 (160.58, 217.58)
	Both	205.84	237.16	174.50	205.84 (174.50, 237.16)

### Age-Period-Cohort analysis of ischemic stroke incidence

The Age-Period-Cohort (APC) model was employed to dissect the trends in ischemic stroke incidence into age, period, and cohort effects, providing insights beyond the crude temporal changes. The detailed data for the APC analysis results are provided in Supplementary Tables 2–6.

#### Age effects

**Figure 2:**
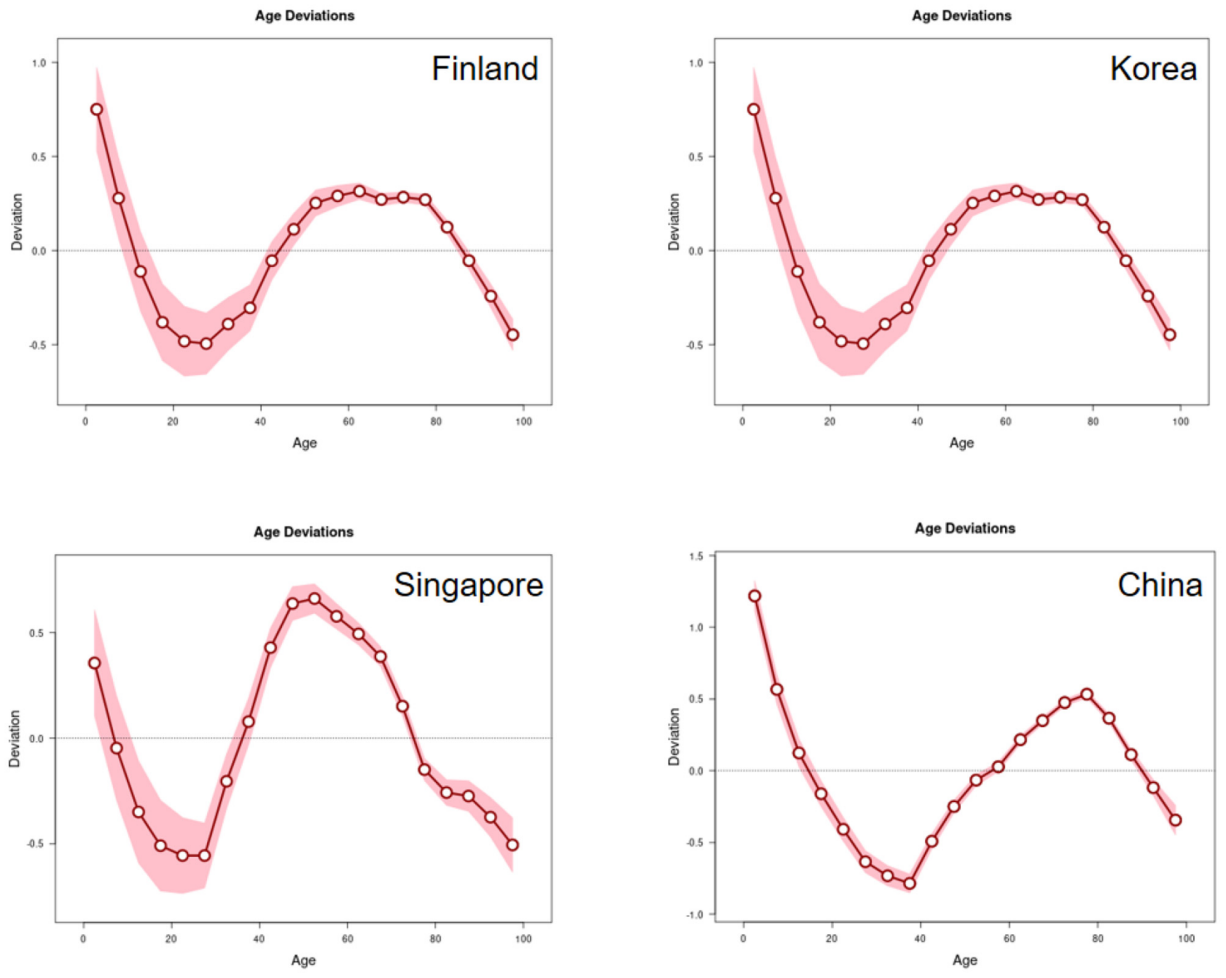
Impact of the age effect on the incidence of IS in Finland, Korea, Singapore and China.

The age effects, which represent the intrinsic risk of disease associated with each age group, exhibited a consistent pattern across Finland, Korea, and Singapore. The relative risk (expressed as logarithmic incidence rate ratios) was significantly below the reference level in the 0–20 age group. This risk increased steadily with age, peaking around the 50-year-old age group. In contrast, China displayed a distinct pattern, where the relative risk remained below the reference level until approximately 40 years of age before rising sharply to peak at around 80 years of age. Moreover, there is a marked trend toward younger age groups experiencing high incidence rates, with the age range shifting from the elderly population (65–94 years) toward middle-aged individuals (40–64 years) (Figure 2). These findings confirm that the biological risk of ischemic stroke is predominantly concentrated in middle-aged and elderly populations in all studied countries.

#### Period effects

**Figure 3:**
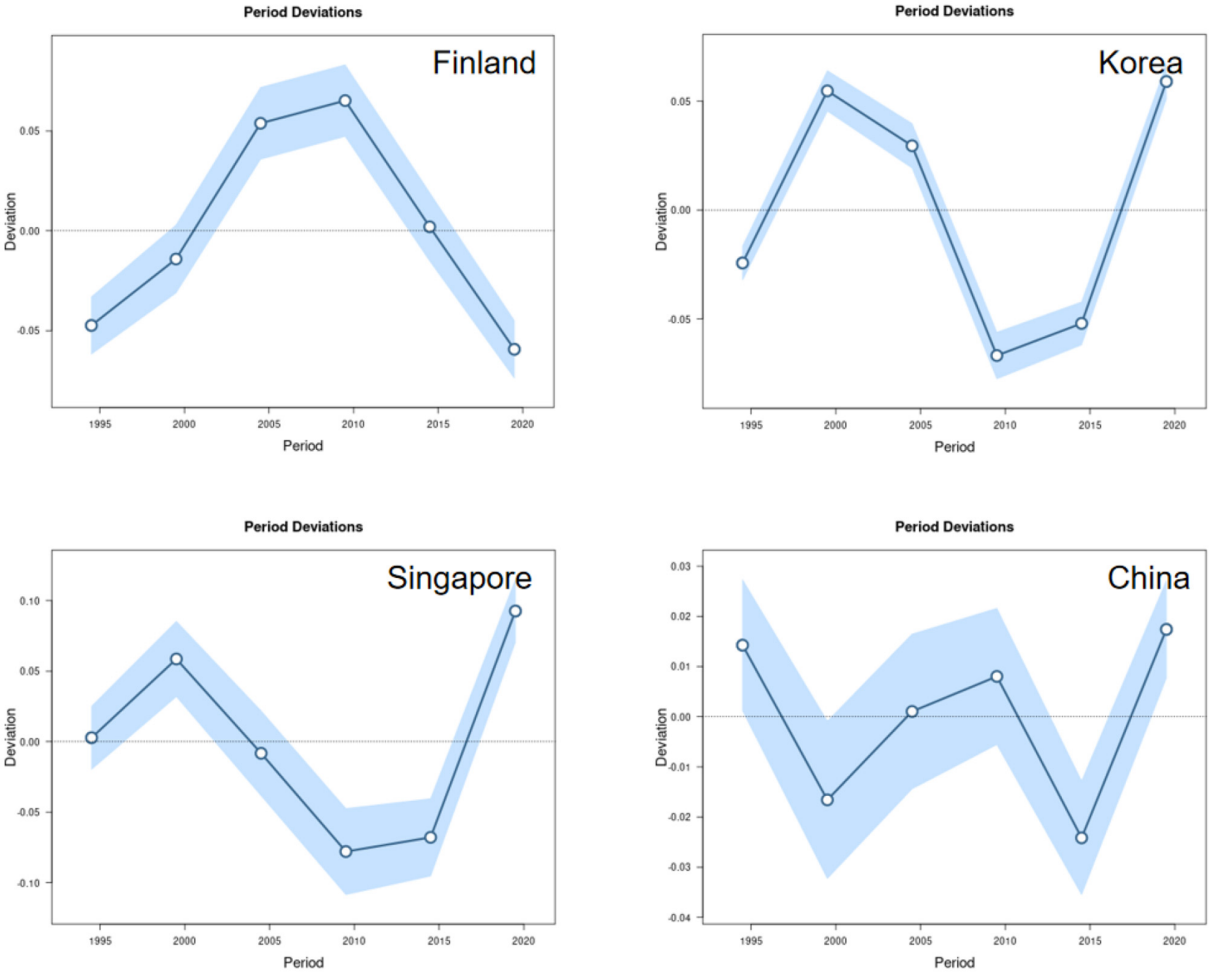
Impact of period effects on the incidence of IS in Finland, Korea, Singapore and China.

The period effects, reflecting the influence of external factors affecting all age groups simultaneously at specific points in time, showed distinct fluctuations across the four countries (Figure 3). A notable increase in relative risk was observed between 1995 and 2000 in Finland, Korea, and Singapore. However, since approximately 2010, only Finland demonstrated a significant declining period effect. In China, the period effects remained relatively stable throughout the study duration, indicating that the incidence rate in China has been less influenced by the broad temporal factors (e.g., population-wide changes in healthcare or policy) that affected the other nations.

#### Cohort effects

**Figure 4:**
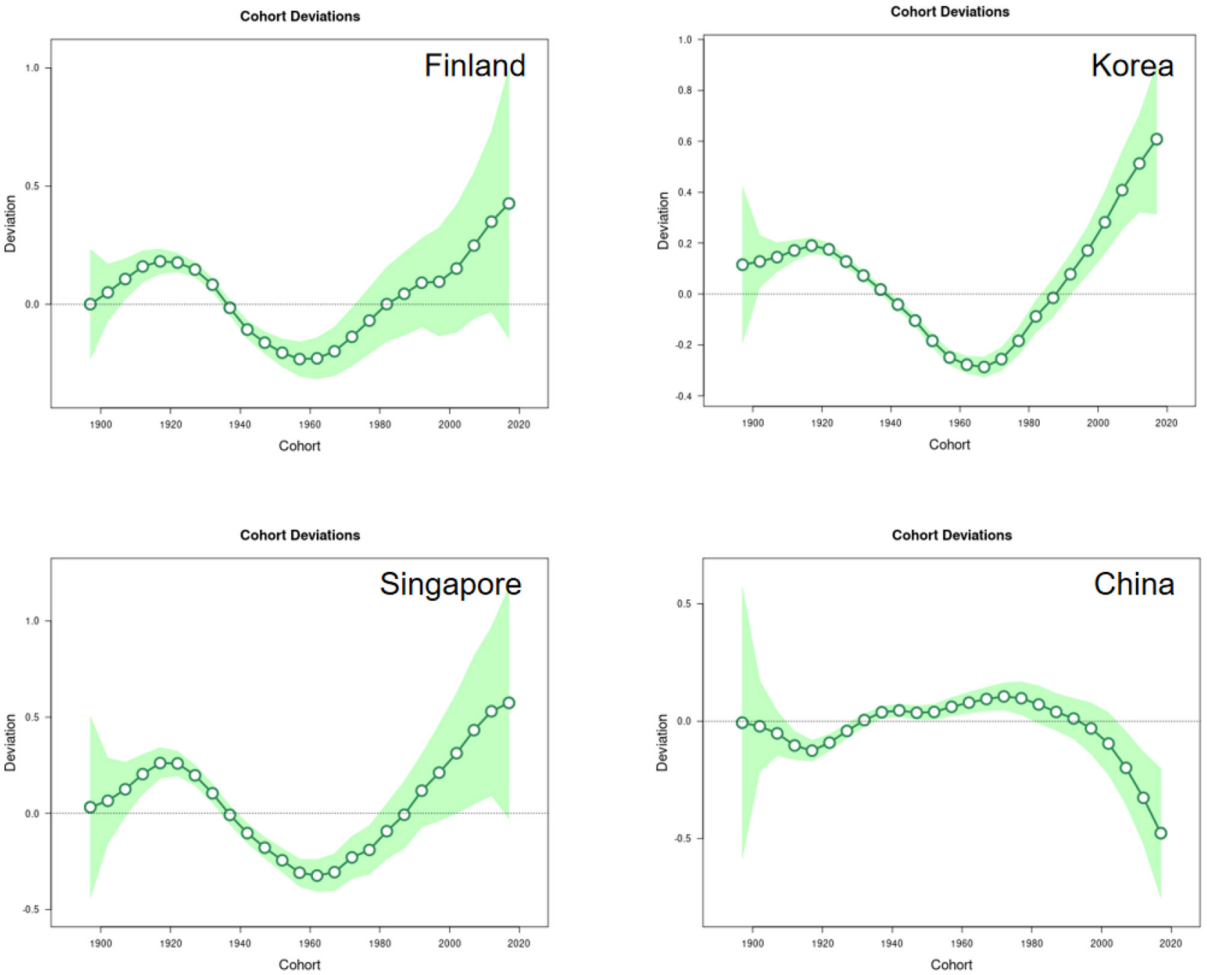
Impact of cohort effects on the incidence of IS in Finland, Korea, Singapore and China.

The cohort effects, indicating the lifetime risk characteristic of specific birth cohorts, revealed important trends in disease risk across generations. The birth cohort deviations for Finland, Korea, and Singapore exhibited a pronounced “U-shaped” pattern. This indicates that earlier and later birth cohorts experience a higher relative risk of ischemic stroke compared to those born in the middle decades (e.g., around 1940–1960) (Supplementary Table 1). Conversely, China's cohort effects showed a generally consistent trend of gradual improvement, with later birth cohorts exhibiting a lower relative risk than their predecessors (Figure 4).

### Future projections of ischemic stroke in 4 countries

**Figure 5:**
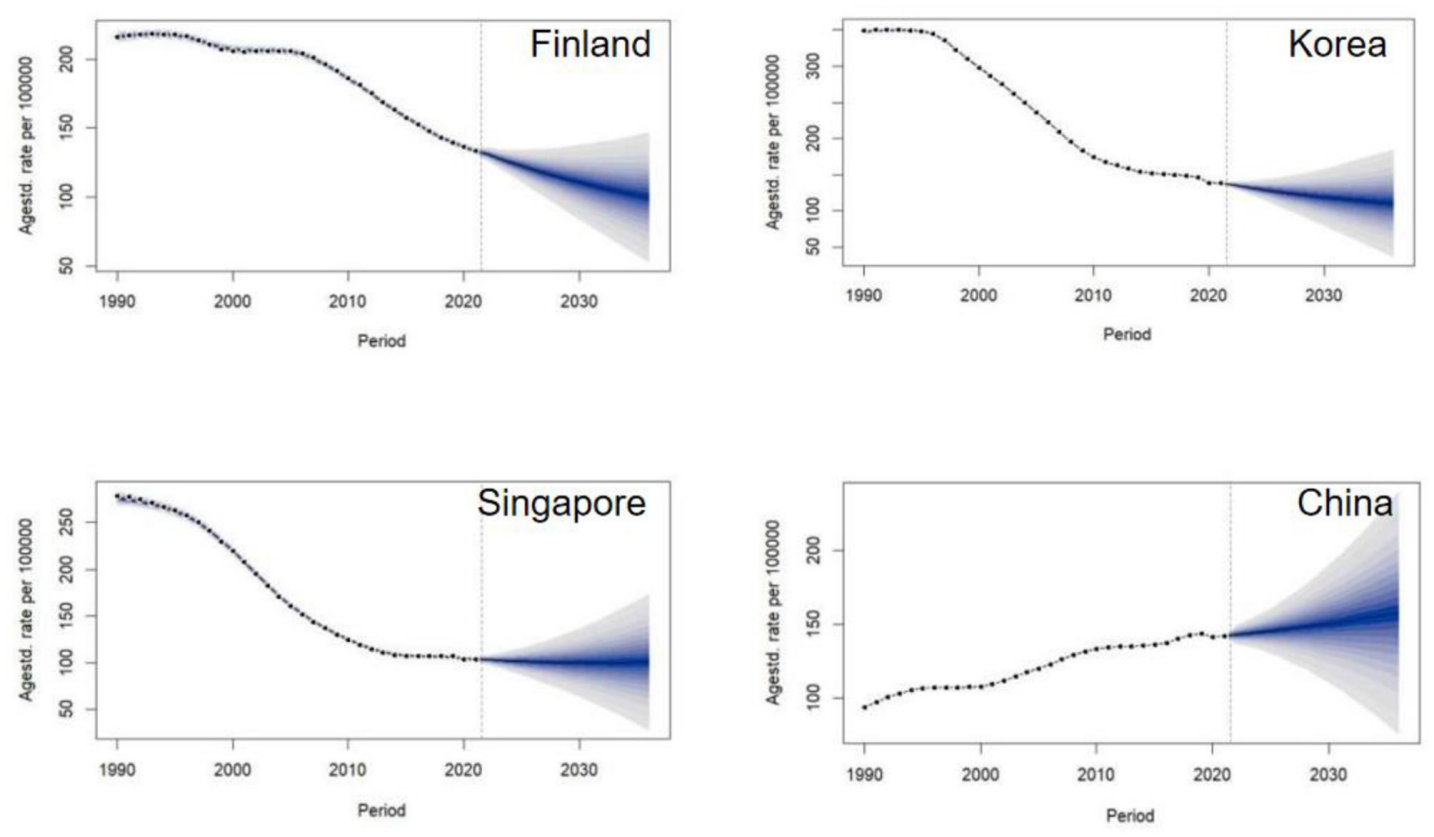
Future projections of ischemic stroke of Finland, Korea, Singapore and China.

The observed trends in age-standardized incidence rates (ASIR) of ischemic stroke from 1990 to 2021, along with projections to 2036, varied substantially among the countries (Figure 5).

Finland: The ASIR displayed a steady decreasing trend from nearly 200 to approximately 100 per 100,000 people. Projections indicate that this decline is plateauing, with the future rate stabilizing around 100 per 100,000. The uncertainty interval for this projection was relatively narrow (95% UI: 40.12 to 119.59), suggesting stable and reliable estimates.

Korea and Singapore: Both countries experienced a dramatic decline in ASIR between 1990 and 2010. In Korea, the rate fell from 300 to below 150 per 100,000, while in Singapore, it decreased from 250 to about 100 per 100,000. Projections for both nations suggest that this downward trend has halted, with incidence rates stabilizing near 100 per 100,000.

China: In contrast, the ASIR in China showed a gradual but persistent increase from about 100 to nearly 150 per 100,000. Projections indicate a continuation of this upward trajectory, with the rate potentially reaching 200 per 100,000 by 2036. The uncertainty interval for this projection was wide (95% UI: 131.29 to 348.36), indicating greater uncertainty in future estimates.

## Discussion

This study utilized the Global Burden of Disease (GBD) database to analyze temporal trends in the incidence, prevalence, and disability-adjusted life years (DALYs) of ischemic stroke in Finland, South Korea, Singapore, and China between 1990 and 2021. It employed age-period-cohort (APC) models to explore age, period, and cohort effects, and utilized Bayesian age-period-cohort (BAPC) models to forecast future incidence trends over the next 15 years. Results indicate that China bears a significantly higher stroke burden than other nations, with incidence rates exhibiting a persistent upward trajectory. Conversely, Finland, South Korea, and Singapore demonstrate relatively lower burdens, characterized by declining or stable incidence trends. Furthermore, age effects reveal a marked increase in ischemic stroke incidence with advancing age, alongside a trend toward younger age groups in China. Period and cohort effects further elucidate the dynamic shifts in stroke burden across countries and their potential drivers.

Although the global average age-standardized incidence rate declined during this period, it has rebounded in recent years and is projected to continue rising over the next 15 years. This reversal may partly relate to rapid global economic development, which has substantially enhanced healthcare standards and public health awareness in some nations. This progress enables governments to establish comprehensive healthcare systems and implement effective public health interventions, such as risk factor management, early disease screening, and prevention (23, 24). However, the global rebound may be linked to population aging, increased chronic disease burden, and changing lifestyles (25–28). Ischemic stroke is a complex cerebrovascular disorder whose pathophysiological mechanisms primarily involve reduced cerebral blood flow, vascular endothelial dysfunction, inflammatory responses, thrombosis, and neuronal injury (29). Research indicates that regional disparities in stroke burden may be closely linked to the prevalence of these mechanisms and the distribution of associated risk factors. For instance, persistent exposure to high-salt diets, high smoking rates, and inadequate management of hypertension are all factors strongly associated with increased risks of vascular endothelial dysfunction and thrombosis (30–33). Moreover, aging is a significant driver of rising stroke incidence, with rapid population aging potentially exacerbating the stroke burden further (34).

Concurrently, substantial variations between nations underscore the complexity of the epidemiological profile of ischemic stroke. These disparities across countries and regions may stem from differences in socioeconomic development, public health policies, cultural practices, and lifestyles. Nations should enhance collaboration and exchange experiences in prevention and treatment to mitigate the multidimensional burden of this disease.

By analyzing the age-period-cohort model, we can further explore the specific reasons for these differences.

From an age perspective, global averages and distribution curves across four countries reveal that the incidence of ischemic stroke increases significantly with age, rising from around 20 years and peaking around 60 years. Concurrently, the elderly population exhibits the highest age-standardized prevalence rates, age-standardized incidence rates, and age-standardized disability-adjusted life years (DALYs). This phenomenon aligns with established associations between age and ischemic stroke risk (35).

The heightened disease burden among the elderly may stem from the cumulative effects of long-term risk factors and the gradual manifestation of degenerative changes within the cerebrovascular system. The most characteristic pathological alterations include reduced arterial wall elasticity and impaired vascular endothelial cell regulatory function (36, 37).

Moreover, in developed nations, the age range for high incidence of ischaemic stroke has progressively shifted from the traditional elderly cohort (65–94 years) toward middle-aged individuals (40–64 years). This trend likely arises from increasing exposure among middle-aged adults to risk factors historically associated with older age groups, such as hypertension, coronary heart disease, diabetes mellitus, hypercholesterolemia, and impaired glucose tolerance (38). Addressing this trend, future research should further analyze specific drivers in the middle-aged population (e.g., hypertension control and lifestyle modifications) and incorporate scenario-based predictive analyses with covariates (e.g., smoking prevalence, dietary patterns, physical activity levels) to generate more policy-relevant outcomes. Concurrently, enhanced early screening and health education for middle-aged individuals should be prioritized, alongside lifestyle interventions (such as exercise and healthy diets) to effectively reduce stroke risk.

From a period perspective, economic globalization has facilitated the dissemination of advanced medical technologies and health-conscious lifestyles, contributing to an overall downward trend in incidence rates. However, fluctuations in period bias curves across nations may correlate with economic factors and public health events; for instance, financial crises may induce heightened stress and deteriorating lifestyles, thereby elevating stroke risk (39–42). Moreover, public health crises (such as the COVID-19 pandemic) may further exacerbate ischemic stroke risk by altering population behaviors (e.g., increased sedentary lifestyles, dietary deterioration). These period effects underscore the necessity for health policy implementation to account for socioeconomic shifts impacting population health.

Notably, declining ischemic stroke incidence in certain countries correlates closely with specific public health policies. For instance, China's incidence reduction between 1995 and 2000 may relate to the successful implementation of national salt reduction campaigns and health education initiatives (43); while Finland's decline since 2010 may be closely linked to reduced smoking rates (44). Future research should further explore the long-term effects of these policy drivers and conduct scenario-based predictive analyses incorporating relevant covariates (e.g., hypertension, diabetes, air pollution) to generate more policy-relevant estimates. Furthermore, it is recommended to assess the calibration and uncertainty of predictive models through post-prediction and a posteriori forecasting checks, thereby enhancing the precision and efficacy of policy interventions.

From a cohort perspective, cohort bias exhibits significant variations across nations. Finland, South Korea, and Singapore demonstrate a characteristic “U-shaped” trend in cohort bias, where bias initially declines before increasing. This pattern reflects the combined effects of healthcare standards, economic and social development, and population aging on incidence rates. South Korea's increasing cohort bias may correlate with its advancing population aging (45). In contrast, China's cohort bias exhibits an overall trend of gradual improvement, indicating that public health interventions and rising living standards positively contribute to reducing ischemic stroke risk (46).

Regarding cohort effects, future research should further explore differences in health behaviors, healthcare resource accessibility, and socioeconomic status across cohorts (47–51). For instance, more in-depth analysis could be conducted on health behaviors (such as dietary patterns and exercise habits) and the distribution of healthcare resources within urban and rural cohorts (28, 30, 52). Furthermore, it is recommended to integrate data on specific subtypes of ischemic stroke (29) to examine the long-term impact of different cohorts on future disease burden, thereby providing more targeted evidence for policy formulation.

Future projections indicate that incidence rates in Finland and Singapore will continue to decline and remain at low levels, whereas China may experience a gradual increase due to population aging and rising chronic diseases (53). The growing burden of chronic conditions such as hypertension and diabetes are likely to be a primary driver of rising ischemic stroke incidence in China (54). In response to this trend, it is recommended to strengthen comprehensive chronic disease management, including multidisciplinary collaboration and community health services (55–57). Furthermore, health education for the elderly population should be enhanced to encourage healthy behaviors and regular medical check-ups. International cooperation also holds significant importance in reducing the burden of ischemic stroke. By sharing successful experiences (such as Finland's smoking rate control and Singapore's health management) (58, 59), countries can collectively address the challenges posed by the burden of ischemic stroke.

This Global Burden of Disease (GBD) study offers a valuable macro-level assessment of the global ischemic stroke burden. By integrating diverse data sources and employing rigorous modeling, it illuminates overall trends and key risk factors, providing a crucial evidence base for understanding global disease patterns and filling knowledge gaps, especially in data-scarce regions. However, the findings must be interpreted with caution due to several limitations.

These limitations primarily lie in three areas. First, the heavy reliance on heterogeneous secondary data may lead to underestimation of the true burden in low- and middle-income countries and introduce compositional bias. The modeling approach itself, while advanced, depends on assumptions and statistical imputation, introducing uncertainty. The absence of sensitivity analysis further limits the ability to assess the robustness of the estimates (2). Secondly, the analysis lacks depth and specificity; it cannot classify strokes into etiological subtypes (e.g., TOAST criteria), examine the compounding effects of risk factors, or account for the influence of existing national stroke prevention and management strategies (5). Thirdly, the study period coincides with the COVID-19 pandemic, which likely confounded trends through increased thrombotic events and widespread healthcare disruptions.

In conclusion, while this study provides an essential macro-perspective on long-term, large-scale trends, its limitations necessitate cautious interpretation of specific regional or national estimates. Future research should aim to incorporate higher-quality primary data, conduct subtype-specific analyses, perform sensitivity analyses, and integrate assessments of healthcare policies. Additionally, moving beyond the current model's projections, which may not capture future disruptive events, by leveraging individual-level data and alternative modeling techniques (e.g., machine learning) would be valuable for exploring specific drivers (e.g., socioeconomic factors, biomarkers) and informing targeted interventions.

## Conclusions

Although the global average age-standardized incidence rate declined between 1990 and 2021, it has shown an upward trend in recent years and is likely to continue to rise over the next 15 years. Significant differences in age-standardized incidence rates of ischemic stroke between Finland, Korea, Singapore and China may be attributed to differences in the level of economic development, healthcare conditions and demographics of each country. Using an age-period-cohort model, this study provides trends in the incidence of ischemic stroke and provides a basis for the development of targeted prevention and management strategies in each country.

## Data Availability

The original contributions presented in the study are included in the article/Supplementary material, further inquiries can be directed to the corresponding author.
