# Supplementary material for: Age-Period-Cohort analysis and 2036 projections of the burden of ischemic stroke in Finland, Korea, Singapore and China, 1990–2021
Source: Front Neurol. 2025 Nov 3;16:1651799. doi: 10.3389/fneur.2025.1651799 (PMC12620190; doi:10.3389/fneur.2025.1651799)
Supplement: Supplementary file 1 [file Table_1.docx]

Supplementary Table 1

| Cohert | 1 | 2 | 3 | 4 | 5 | 6 | 7 | 8 | 9 |
| --- | --- | --- | --- | --- | --- | --- | --- | --- | --- |
| Birth Year | 1897 | 1902 | 1907 | 1912 | 1917 | 1922 | 1927 | 1932 | 1937 |
| Cohert | 10 | 11 | 12 | 13 | 14 | 15 | 16 | 17 | 18 |
| Birth Year | 1942 | 1947 | 1952 | 1957 | 1962 | 1967 | 1972 | 1977 | 1982 |
| Cohert | 19 | 20 | 21 | 22 | 23 | 24 | 25 |  |  |
| Birth Year | 1987 | 1992 | 1997 | 2002 | 2007 | 2012 | 2017 |  |  |
